# Two Natural *ent*-kauranoids as Novel Wnt Signaling Inhibitors

**DOI:** 10.1007/s13659-014-0016-4

**Published:** 2014-04-24

**Authors:** Jing Zhang, Ling-Mei Kong, Rui Zhan, Zhen-Nan Ye, Jian-Xin Pu, Han-Dong Sun, Yan Li

**Affiliations:** 1 State Key Laboratory of Phytochemistry and Plant resources in West China, Kunming Institute of Botany, Chinese Academy of Science, Kunming, 650201 Yunnan China; 2 University of Chinese Academy of Science, Beijing, 100049 China

**Keywords:** Natural *ent*-kauranoids, Wnt signaling, Inhibitors, Colon cancer

## Abstract

Constitutively active Wnt signaling frequently occurs in most colon cancers. Therefore, inhibitors of Wnt signaling pathway could provide rational therapeutic effects for colorectal malignancy. Within this paper, we identified two inhibitors of Wnt signaling pathway, rabdoternin B and maoecrystal I from a natural *ent*-kauranoid library by a dual-luciferase reporter gene assay. The two compounds inhibited Wnt signaling pathway in a concentration-dependent manner and exhibited selective cytotoxicity toward a number of colon carcinoma cell lines SW480, HCT116, and HT29, with only weak cytotoxicity towards the normal colonic epithelial cell line CCD-841-CoN. Rabdoternin B and maoecrystal I treatment induced G2/M phase arrest efficiently in SW480 cells as revealed by flow cytometry analysis. A further study found that maoecrystal I decreased the expression of Wnt signaling target genes, including *c*-*myc*, *cyclin D1*, *survivin* and *Axin2* in colon cancer cells. Collectively our data suggests that rabdoternin B and maoecrystal I are novel inhibitors of canonical Wnt signaling pathway and may possess potentials for colon cancer therapy.

## Introduction

The canonical Wnt signaling pathway plays a crucial role in tissue development, homeostasis, and cancers [1]. When cells are inactivated, the destruction complex consisting of GSK-3β/Axin2/APC/CK1, phosphorylates β-catenin at the key N-terminal Ser and Thr residues in the cytoplasm and stimulates its ubiquitination-dependent destruction. Upon binding of Wnt ligands to its receptor complex, the destruction complex disassociates, followed by β-catenin accumulation in the cytoplasm and translocation to the nucleus [2]. In the nucleus β-catenin integrates with transcription factors T cell factor 4/lymphoid enhancer-binding factor 1 (TCF4/LEF1) to initiate transcription of Wnt/β-catenin signaling responsive genes, such as *c*-*myc* and *cyclin D1*, which are key regulators of cell cycle and cell growth.

Colon cancer is a common disease and is one of the leading causes of cancer death [3]. APC (Adenomatous Polyposis Coli) is a core component of the Wnt signaling pathway. Its gene, *APC*, the tumor suppressor gene, mutated in about 80 % colon cancers [4, 5]. One of the first initiating events in colorectal tumorigenesis is believed to be loss of functional APC [6]. Mutations of *β*-*catenin*, *Axin2* and GSK-3β that lead to hyperactive Wnt signaling are also found in colon cancers [7–9]. Therefore, the Wnt/β-catenin signaling pathway has been considered to be a potential target in the treatment of colon cancer. The discovery of compounds targeting Wnt signaling pathway has great potential to become a therapeutic strategy for colon cancer treatment. Several screened small molecules, such as NC043(15-oxospiramilactone) [10], XAV939 [11], ICG001 [12] etc, have been identified to display in vivo and in vitro anti-cancer activity in colon cancer, probably through affecting the β-catenin/TCF4 association, tankyrase activity, and connection of β-catenin and CREB-binding protein, respectively.

**Fig. 1:**
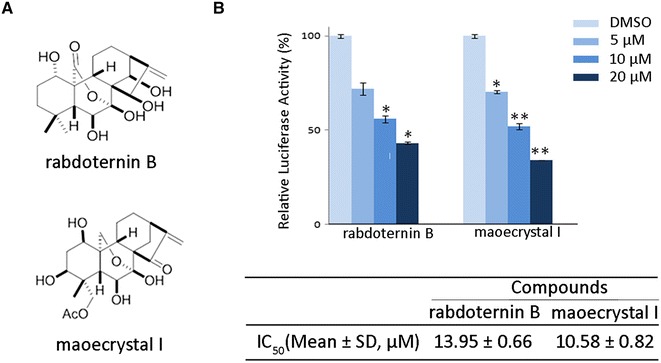
Rabdoternin B and maoecrystal I are two novel inhibitors of Wnt signaling pathway. **a** The chemical structures of rabdoternin B and maoecrystal I. **b** Rabdoternin B and maoecrystal I inhibit luciferase activity in a concentration-dependent manner in HEK293W cells. HEK293W cells were treated with rabdoternin B and maoecrystal I at indicated dosages for 24 h respectively and the luciferase activity was measured. Data represents the mean ± SD (*error bars*) from three independent experiments. Statistical significance was examined by paired-*t* test. * *p* < 0.05, ** *p* < 0.01

Natural products and their derivates, with their structural diversity, offer a rich resource for identification of novel inhibitors of Wnt signaling. The genus *Isodon* (Labiatae) is widely distributed plants, many of which are used for the treatment of cancer and inflammation in traditional Chinese medicine. Over the past twenty years, the structures and bioactivities of their diterpenoids constituents, especially those with an *ent*-kaurane skeleton, have received considerable phytochemical and biological attention [13]. In the present study, we performed a biological screening for the inhibitors of Wnt signaling from the library of natural *ent*-kaurane diterpenoids with dual-luciferase reporter gene assay [14] and identified rabdoternin B and maoecrystal I as Wnt signaling pathway inhibitors. Rabdoternin B and maoecrystal I (Fig. 1a), two *ent*-kauranoids isolated from the aerial parts of *Isodon rosthornii* [15] and leaves of *Isodon xerophilus* [16], respectively, effectively inhibited Wnt signaling pathway and exhibited preliminarily selective cytotoxicity toward colon carcinoma cells. The identified rabdoternin B and maoecrystal I may serve as an attractive start point for novel colon cancer drug development.

## Results and Discussion

### Rabdoternin B and Maoecrystal I are Two Novel Inhibitors of Canonical Wnt Signaling Pathway

To identify compounds that inhibit the canonical Wnt signaling pathway, two hundred *ent*-kauranoids from a natural compounds library were screened using HEK293 cells that were stably co-transfected with *Topflash, renilla reporters* and *wnt3A*, namely HEK293W cells [14]. Compounds causing more than a 50 % decrease of the relative luciferase activity were considered as “hits”. To avoid “false positive” hits, we assessed the cytotoxicity and excluded the compounds that caused more than 50 % decrease of the value of Renilla, compared with control. The screening resulted in about 5 % primary hits. To determine the 50 % inhibitory concentrations (IC_50_) of these primary hits on Wnt signaling pathway, we performed secondary screening using three concentrations by two-fold dilution, with the highest concentration being 20 μM. As shown in Fig. 1b, rabdoternin B and maoecrystal I concentration-dependently inhibited the canonical Wnt signaling in HEK293 W cells, with IC_50_ values of 13.95 ± 0.66 µM and 10.58 ± 0.82 µM, respectively.

### Rabdoternin B and Maoecrystal I Exhibit Cytotoxicity in Colon Cancer Cells

**Fig. 2:**
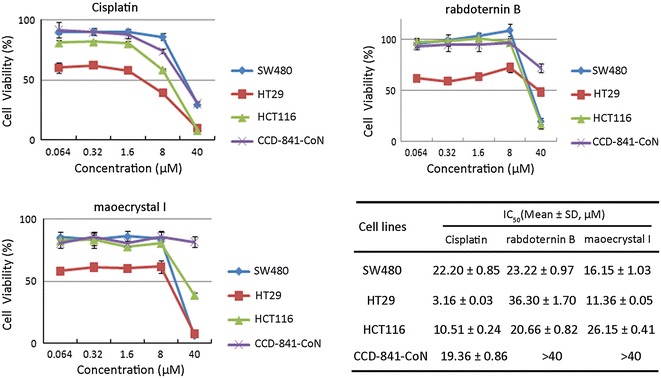
Rabdoternin B and maoecrystal I inhibit the growth of SW480, HT29 and HCT116 cells. Cells were treated with indicated concentrations of compounds for 48 h, and cell viability was assessed using CellTiter 96 Aqueous one solution cell proliferation assay. Data represents mean ± SD of three independent experiments

It is well known that Wnt signaling plays a critical role in regulation of cell growth, proliferation, and survival. Constitutively active Wnt signaling are found to be involved in about 90 % colon cancer cells [17]. Moreover, inhibitors of Wnt signaling are supposed to abrogate the growth and survival of cancer cells. Therefore, the cytotoxic effects of rabdoternin B and maoecrystal I were investigated by MTS assay in three colon cancer cell lines, SW480 and HT29, with *APC* mutated [18], and HCT116 with deletion of *β*-*catenin* on site S45 [19], which are all known to be Wnt signal over-activated cells, with a normal colonic epithelial cell line CCD-841-CoN as control. As shown in Fig. 2, rabdoternin B and maoecrystal I significantly decreased the cell viability of all the three colon cancer cell lines, with only slight toxicity conferred towards normal colon cells, whereas cisplatin (DDP), the commonly used drug for cancer treatment, showed general cytotoxicity toward all the cell lines tested. The cytotoxic selectivity toward colorectal cancer cells that rabdoternin B and maoecrystal I exhibited preliminarily, make them both promising candidates for further anti-cancer agent development.

### Rabdoternin B and Maoecrystal I Induce Cell Cycle Arrest in Colon Cancer Cells

**Fig. 3:**
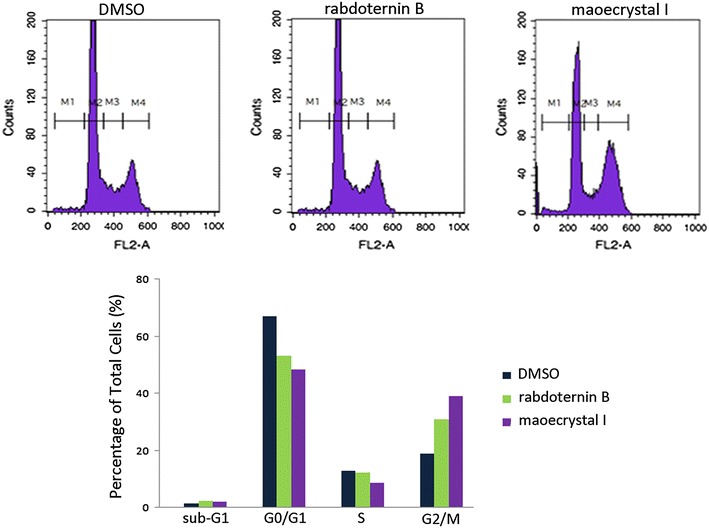
Rabdoternin B and maoecrystal I arrest cell cycle at G2/M phase in SW480 cells. SW480 cells were incubated with rabdoternin B and maoecrystal I at indicated concentrations of compounds for 48 h respectively. Then cells were stained with PI and analyzed on FACS Calibur. Representative data of three independent experiments was shown

As Wnt signaling plays an important role in the regulation of cell cycle, we measured the effects of rabdoternin B and maoecrystal I on the cell cycle of colon cancer cells. As shown in Fig. 3, treatment of rabdoternin B and maoecrystal I (20 μM) toward SW480 cells for 48 h, respectively, dramatically arrested cells at the G2/M phase, which indicates that rabdoternin B and maoecrystal I, may inhibit the growth of tumor cells through inducing cell cycle arrest. Compared with rabdoternin B, maoecrystal I displayed a more potent effect on cell cycle arrest.

Several studies demonstrated that Wnt signaling influences cell cycle by the up-regulation of G1 effectors on both a transcriptional and translational level [20]. However, Wnt signaling also modulates mitosis. A study demonstrated that SW480 cells contain a relatively higher level of Axin2, with a weak mitotic spindle checkpoint [21]. In addition, Axin2, the Wnt target gene, localizes at mitotic spindles and centrosomes in colon cancer cells. Knockdown of *Axin2* in SW480 cells and knockout of *Axin2* in mouse embryo fibroblasts (MEF) cells both enhanced the fraction of cells at the G2/M phase. Moreover, there is a hypothesis that Axin2, as a signaling, modulates the mitotic spindle checkpoint [22]. Our results demonstrated that G2/M phase arrest of SW480 cells induced by rabdoternin B and maoecrystal I could be due to the inhibition of Wnt signaling.

### Maoecrystal I Inhibits Endogenous Wnt Signaling Pathway in Colon Cancer Cells

**Fig. 4:**
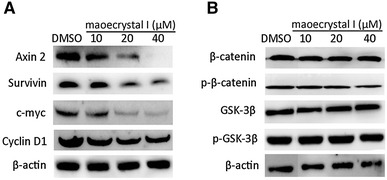
Maoecrystal I downregulates expression of endogenous Wnt target genes without affection on β-catenin stability. **a** Maoecrystal I downregulates endogenous Wnt target genes in SW480 cells in a dose-dependent manner. SW480 cells were treated with 0, 10, 20, and 40 μΜ maoecrystal I for 24 h and cell lysates were subjected to Western blotting with antibodies for c-Myc, cyclin D1, survivin and Axin 2, with β-actin as loading control. **b** Maoecrystal I does not affect β-catenin stability. SW480 cells were treated with 0, 10, 20, and 40 μΜ maoecrystal I for 24 h and cells lysates were subjected to Western blotting analysis. β-actin was used as loading control

Since rabdoternin B and maoecrystal I were identified to be inhibitors of Wnt signaling, we wanted to identify if the inhibition of cancer cell growth by the two compounds is due to the inhibition of Wnt signaling. Compared with rabdoternin B, maoecrystal I exhibited more potent cytotoxic effects and more remarkable cell cycle arrest activity in colon cancer cells, therefore, we explored the effects of maoecrystal I on endogenous Wnt signaling pathway in colorectal cancer cells. *C*-*myc*, *cyclin D1*, *survivin*, and *Axin2* are well-known target genes of Wnt signaling pathway [23–26]. The expressions of the endogenous Wnt target genes were tested in SW480 cells treated with maoecrystal I by using Western blotting analysis. As shown in Fig. 4a, treatment of SW480 cells with increasing concentrations of maoecrystal I for 24 h reduced expressions of *c*-*myc*, *cyclin D1*, *survivin*, and *Axin2* in a dose-dependent manner, which showed that the inhibition of cancer cell growth is associated with Wnt signaling inhibition by maoecrystal I.

Regulation of β-catenin level is a feature of Wnt signaling pathway. Wnt/β-catenin signaling is activated after β-catenin accumulates and translocates to the nucleus and binds to other transcription factors [1]. To investigate the molecular mechanism of maoecrystal I that inhibits Wnt signaling pathway, the protein level of phosphorylated β-catenin and total β-catenin in SW480 cells were tested by Western blotting analysis. As shown in Fig. 4b, maoecrystal I did not change either phospho-β-catenin (S33/37/T45) or the total β-catenin at the protein level. These results suggest that maoecrystal I may interfere with downstream events of β-catenin degradation to inhibit β-catenin/TCF4 transcriptional activity. Moreover, GSK-3β, the kinase that regulates the phosphorylation of β-catenin on site S33/37/T45, and its inactive phosphorylation form, phospho-GSK-3β (S9) [27], were not affected by maoecrystal I treatment either (Fig. 4b).

Regulation of β-catenin to inhibit Wnt signaling pathway usually occurs on two distinctive levels, promoting β-catenin degradation or inhibiting association of β-catenin and transcriptional factors. Our findings reveal that maoecrystal I did not affect total β-catenin at the protein level, suggesting that maoecrystal I has no influence on β-catenin destruction. Therefore, we deduce that maoecrystal I may inhibit Wnt signaling pathway downstream of β-catenin destruction, such as suppressing the translocation of β-catenin into nucleus or reducing the association of β-catenin with transcription factors TCF4/LEF1, two important events that are downstream of β-catenin degradation.

## Conclusion

Two natural *ent*-kauranoids, rabdoternin B and maoecrystal I, as novel inhibitors of Wnt signaling pathway were identified in our study. Both rabdoternin B and maoecrystal I inhibited the growth of colon carcinoma cells and arrested SW480 cells at G2/M phase, which were associated with decreased expression of Wnt target genes. Maoecrystal I did not affect β-catenin level in SW480 cells, suggesting that maoecrystal I might inhibit Wnt signaling though downstream events of β-catenin degradation. In culmination, rabdoternin B and maoecrystal I, as novel inhibitors of canonical Wnt signaling pathway, may possess the potentials for colon cancer therapy.

## General Experimental Procedures

### Materials

SW480, HCT116, HT29, and CCD-841-CoN were obtained from ATCC. RPMI 1640, Dulbecco’s modified Eagle medium (DMEM), fetal bovine serum (FBS), antibiotics–antimycotics solution and trypsin–EDTA were purchased from HyClone (Logan, UT). Mouse monoclonal anti-β-catenin antibody was from BD; Mouse monoclonal anti-Cyclin D1, anti-survivin, anti-β-actin antibodies were from Santa Cruz Biotechnology; Rabbit multiclonal anti-phospho-β-catenin (S33/37/T41), mouse monoclonal anti-GSK-3β, anti-phospho-GSK-3β (S9), rabbit anti-Axin2 antibodies were from Cell Signaling Technology; Hygromycin B, G418 and EGCG were purchased from Sigma. Rabdoternin B and maoecrystal I were isolated from the aerial parts of *Isodon rosthornii* [15] and leaves of *Isodon xerophilus* [16].

### Cell Culture

SW480, HCT116, and CCD-841-CoN were propagated in Dulbecco’s modified Eagle’s medium (DMEM), and HT29 were maintained in modified RMPI-1640 medium, both supplemented with 10 % fetal bovine serum, 100 μg/mL penicillin, 100 μg/mL streptomycin. HEK293W were cultured in DMEM medium, supplemented with 10 % fetal bovine serum, 100 μg/mL penicillin, 100 μg/mL streptomycin, 100 μg/mL G418 and 100 μg/mL Hygromycin B. Cells were maintained with 5 % CO_2_ in a humidified incubator at 37 °C.

### MTS Cell Viability Assay

100 μL cells were seeded in 96-well plate with a density of 5000 cells per well the day before compounds treatment. Cells were incubated with test compounds with the concentration range 0.064–40 μΜ for 48 h in triplicates. Then cell viability was evaluated using CellTiter 96 Aqueous One Solution Cell Proliferation Assay (Promega) according to the manufacturer’s instructions. The absorbency of each well (OD_490 nm_ value) was measured using microplate reader (Bio-Rad). Cell viability was determined and the 50 % inhibitory concentrations (IC_50_) were calculated by Reed and Muench method.

### Dual-luciferase Reporter Gene Assay

A stable transfected human kidney epithelial cell line HEK293W was created as described and used for assay of inhibitory effect of compounds on Wnt signaling [14]. 100 μL cells were plated in 96-well plate with a density of 5000 cells per well. After 12 h, compounds were added and incubated for further 24 h. Luciferase activity was measured using the Dual-Lucy Assay Kit (Promega) as manufacture’s protocol. The medium was discarded and 50 μL 1× lysis buffer was added to each well. After 30 min vibration, with 1× substrate and stop buffer each prepared, luciferase activity was measured by Ascent software on chemiluminescent plate reader (Thermo). Data was analyzed and the 50 % inhibitory concentrations (IC_50_) were calculated by Reed and Muench method.

### Cell Cycle Analysis

SW480 cells were plated in 6-well plates and incubated overnight at 37 °C. Rabdoternin B and maoecrystal I were added, respectively, and the cells were incubated for further 48 h. Cells were then collected and fixed in pre-cooled 70 % ethanol at −20 °C for 1 h. After washing with PBS, RNase (25 μg/mL) was added for 30 min at 37 °C for digestion, and then stained with PI (100 μg/mL) in dark for 20 min. Fluorescence intensity was analyzed by FACS Calibur flow cytometer (BD Biosciences, San Jose, CA, USA). The percentages of the cells distributed in different phases of the cell cycle were determined using ModFIT LT 2.0.

### Western Blotting

Cells were seeded in 6-well plates and incubated at 37 °C overnight. Total cell lysates were prepared by direct lysis on ice in lysis buffer (62.5 mM Tris-HCl, pH 6.8, 2 % SDS, 10 % glycerol, 5 % β-mercaptoethanol, and 0.02 % bromophenol blue) after compounds treatment. Samples were then fractionated on 10 % acrylamide gel, transferred to a PVDF membrane (Bio-Rad), and incubated with specific primary antibodies followed by the corresponding peroxidase-conjugated secondary antibodies. Proteins of interest were visualized by chemiluminescent detection on an ImageQuant LAS mini4000 (GE Healthcare).

### Statistical Analysis

Statistical analysis was performed via paired-*t* test using Excel 2007 software. In all the assays, the probability value (*p*) of less than 0.05 was considered as statistically significant.
